# Retrospective Study on the Features and Outcomes of a Tuscany COVID-19 Hospitalized Patients Cohort: Preliminary Results

**DOI:** 10.3390/jcm13164626

**Published:** 2024-08-07

**Authors:** Caterina Silvestri, Cristina Stasi, Francesco Profili, Simone Bartolacci, Emiliano Sessa, Danilo Tacconi, Liliana Villari, Laura Carrozzi, Francesco Dotta, Elena Bargagli, Sandra Donnini, Luca Masotti, Laura Rasero, Federico Lavorini, Francesco Pistelli, Davide Chimera, Alessandra Sorano, Martina Pacifici, Caterina Milli, Fabio Voller, SPRINT Study Group

**Affiliations:** 1 Epidemiology Unit, Regional Health Agency of Tuscany, 50141 Florence, Italy; 2 Department of Medicine, Surgery and Neuroscience, University of Siena, 53100 Siena, Italy; 3 Infectious Diseases Unit, PO San Donato, 52100 Arezzo, Italy; 4 Division of Pneumology, AUSL Toscana Nord-Ovest, Apuane Hospital, 54100 Massa, Italy; liliana.villari@uslnordovest.toscana.it; 5 Pneumology Unit, Pisa University Hospital, 56124 Pisa, Italy; 6 Diabetes and Metabolic Diseases Unit, Azienda Ospedaliera Universitaria Senese, 53100 Siena, Italy; 7 Respiratory Diseases Unit, Department Medical Sciences, Surgery and Neurological Sciences, Siena University, 53100 Siena, Italy; 8 Department of Life Sciences, University of Siena, 53100 Siena, Italy; 9 Internal Medicine II and Stroke Unit, San Giuseppe Hospital, 20123 Empoli, Italy; 10 Department of Health Sciences, Clinical Innovations and Research Unit, Careggi University Hospital, University of Florence, 50121 Florence, Italy; 11 Department of Experimental and Clinical Medicine, University of Florence, Largo Brambilla 3, 50134 Florence, Italy; federico.lavorini@unifi.it (F.L.);

**Keywords:** COVID-19, SARS-CoV-2, hospitalization, liver, AST, ALT, NIV

## Abstract

**Background:** A few months after the COVID-19 pandemic onset, knowledge of SARS-CoV-2 infection and outcomes and treatments blew up. This paper aimed to evaluate the features of a Tuscany COVID-19 hospitalized cohort and to identify risk factors for COVID-19 severity. **Methods:** This retrospective observational COVID-19 cohort study (1 March 2020–1 March 2021) was conducted on patients ≥ 18 years old, admitted to Tuscany Hospital, and subjected to follow-up within 12 months after discharge. Patients were enrolled at Pisana, Senese and Careggi University Hospitals, and South East, North West, and Center Local Hospitals. **Results**: 2888 patients (M = 58.5%, mean age = 66.2 years) were enrolled, of whom 14.3% (N = 413) were admitted to an intensive care unit. Smokers were 25%, and overweight and obese 65%. The most used drugs were corticosteroids, antacids, antibiotics, and antithrombotics, all antiviral drugs, with slight differences between 2020 and 2021. A strong association was found between outcomes of evolution towards critical COVID-19 (non-invasive mechanical ventilation (NIV) and/or admission to intensive care) and smoking (RR = 4.91), ex-smoking (RR = 3.48), overweight (RR = 1.30), obese subjects (RR = 1.62), comorbidities (aRR = 1.38). The alteration of liver enzymes (aspartate aminotransferase, alanine aminotransferase, or gamma-glutamyl transpeptidase) was associated with NIV (aOR = 2.28). **Conclusions:** Our cohort, characterized by patients with a mean age of 66.2 years, showed 65% of patients were overweight and obese. Smoking/ex-smoking, overweight/obesity, and other comorbidities were associated with COVID-19 adverse outcomes. The findings also demonstrated that alterations in liver enzymes were associated with worse outcomes.

## 1. Introduction

SARS-CoV-2 infects cells expressing angiotensin-converting receptor 2 (ACE2) and transmembrane serine protease 2 (TGRBSS2), which are expressed in the respiratory mucosa, gastrointestinal tract, and also in the heart and kidney [1]. SARS-CoV-2 uses a spike glycoprotein (s protein) to bind to the ACE2 receptor. Upon binding, the serine protease TGRBSS2 provides the virus entry into the host cell, where the virus replicates. The clinical syndrome caused by the virus is coronavirus disease 2019 (COVID-19). Like other viruses, especially RNA ones, SARS-CoV-2 also constantly evolves through mutations in the genome. Such mutations have been observed around the world since the beginning of the pandemic. Although some of these mutations did not have a significant impact, some led to several variants of concern (VOCs), with them spreading in waves. VOCs present different degrees of transmissibility, greater pathogenicity with more severe forms of disease, or the possibility of evading previously acquired immunity through natural infection or vaccination [2].

A recent study has highlighted that severe forms of COVID-19 are characterized by a clonal expansion of CD8+ T lymphocytes and an increase in the ratio between effector CD8+ T cells and memory T cells, while a mild form of the disease is characterized by the presence of circulating follicular helper T cells. This highlights how the immune response contributes to the pathogenesis of COVID-19 [3]. Following SARS-CoV-2 infection, some infected individuals may remain asymptomatic or exhibit only mild upper respiratory symptoms, while others develop pneumonia and acute respiratory distress syndrome (ARDS), requiring intensive care intubation and presenting complications with a fatal outcome. Given the lack of an etiopathogenetic therapy, based on the pathological characteristics and COVID-19 different clinical stages, the classes of drugs used, in particular in patients with moderate to severe COVID-19, have been antiviral agents (used for other viral pathologies, such as the case of Remdesivir [4,5], initially developed for the treatment of Ebola), monoclonal antibodies, inflammation inhibitors/antirheumatic drugs, and low molecular weight heparins [6].

In a context where information regarding the progression and treatment of COVID-19 was still very uncertain, the main scientific societies began to publish the first documents regarding the follow-up of long-term COVID-19 [7]. In 2020, the British Thoracic Society published the Guidance on Respiratory Follow-Up of Patients with a Clinico-Radiological Diagnosis of COVID-19 Pneumonia, which diversified follow-up protocols based on the severity of the clinical picture [7]. Puntmann et al. [8] highlighted the need to activate cardiac monitoring on patients recovered from COVID-19.

Even in Italy, the first monitoring studies revealed a high percentage (87.4%) of recovered patients with persistent symptoms [9], in particular, a sense of fatigue and dyspnoea, highlighting the need to collect information on the history of symptoms and their severity during the acute phase of COVID-19.

Although in Tuscany the monitoring of the daily progress of cases of SARS-CoV-2 infection represented a fundamental information asset to plan the services necessary for the care of these patients, the data analyzed did not contain information capable of stratifying the risk of susceptibility to infection and the different degree of severity of COVID-19.

To define a homogeneous follow-up procedure within the regional territory capable of evaluating the possible clinical evolution of the SARS-CoV-2 infection, the Regional Health Agency of Tuscany, in collaboration with University Hospitals of Tuscany and the operational units of the Local Hospitals, promoted an observational cohort study entitled “Prospective and retrospective study on the outcomes and complications of COVID-19 in a cohort of hospitalized patients in Tuscany (SPRINT)”.

Therefore, the endpoints of this paper focus on preliminarily evaluating the clinical features of a COVID-19 surviving cohort of people admitted to the hospital in 2020 and 2021 and identifying the factors capable of influencing the severity (admission to an intensive care unit and NIV) and prognosis of patients hospitalized in Tuscany for SARS-CoV-2 infection.

## 2. Study Design

In May 2020, Tuscany Region, with executive decree 7731/2020, approved the “Call COVID-19 research Tuscany”. The project entitled “Prospective and Retrospective Study on the Outcomes and Complications of COVID-19 in a cohort of hospitalized patients in Tuscany” won the call.

This is a retrospective observational cohort study carried out on a population aged ≥ 18 years discharged (discharge date 1 March 2020–1 March 2021) from hospital facilities in Tuscany with a COVID-19 diagnosis, subjected to follow-up. The patients were enrolled in the Pisana, Senese and Careggi University Hospitals, and North West, South East, and Center local hospitals within the follow-up phase. They gave their consent to access the data collected during the hospitalization. The subjects enrolled at admission who did not follow up were excluded from the study. Therefore, the entire cohort consisted of patients hospitalized in the index period with at least one follow-up visit in the 12 months following discharge. The COVID-19 diagnosis was selected using the ICD-9-CM codes indicated in the Decree of the Ministry of Health of 28 October 2020: “Integration of the classification systems adopted for the coding of the clinical information contained in the hospital discharge form and for the remuneration of hospital services as a result of the new SARS-CoV-2 disease (COVID-19). Amendments to the decree of 18 December 2008. (21A00441)”. Using a universal code attributed by Region Tuscany to each person, these patients were matched with hospital admission codes identified by the International Classification of Diseases, Ninth Revision (ICD-9) Clinical Modification. Comorbidities were analyzed, using ICDIX-Cm codes, in secondary diagnoses at discharge. To estimate the frailty, a range of comorbidities was analyzed [10]. The main information collected during the study period is summarized in Table 1.

Since it deals with sensitive data, the study was submitted to and obtained a favorable opinion from the Regional Ethics Committee (EC) and the company task forces (Prot.17508). All participants provided their consent to participate.

In the hospitalization phase, the survey times were identified as T0 (hospital admission) and then every 5 days from the date of hospitalization, considering the day of discharge as the last data collection (regardless of the time elapsed since the previous data entry). In this study, we considered the T0 and the discharge data.

Each test was classified as positive if outside the threshold. When the cut-off values were different between the hospitals involved in the study, the statistician (FP) calculated the positivity based on the specific cut-offs considered.

Data on liver enzymes were recorded only at the University Hospital of Pisa; therefore the cut-offs applied at this hospital were used for subsequent analyses. Patients were grouped based on the presence of at least one of the liver enzymes (aspartate aminotransferase, AST and/or alanine aminotransferase, ALT and/or gamma-glutamyl transpeptidase, gammaGT) outside the normal range.

The group with a more severe form of COVID-19 consisted of patients undergoing NIV, used as the primary modality of respiratory support and admission to the intensive care unit.

The staging of COVID-19 pneumonia was carried out considering the phases of evolution of the clinical picture as early, mild, and severe, according to the classification reported in D’Andrea et al., 2020 [11]. According to the World Health Organization (WHO), patients who underwent non-invasive mechanical ventilation and those admitted to an intensive care unit were considered affected by critical COVID-19 [12].

### 2.1. Data Management

The Clinic Operational Unit involved (both during hospitalization and follow-up) drew up the list of patients enrolled in the study. In the case of computerized medical records, they proceeded to ask the Operational Unit of Computerization of the healthcare processes of our company or to ESTAR the extraction of the fields required by the protocol and the subsequent pseudonymization via IDUNI (hospitalization and follow-up). In the case of non-computerized medical records or which do not provide for individual data extraction, the Regional Health Agency of Tuscany (ARS) has provided each Operational Unit with a web-oriented computerized archive containing all the fields required by both the hospitalization and follow-up protocol. In addition, in this case, the clinician sent the list of enrolled patients to the health authority (or ESTAR) to proceed with the pseudonymization of the demographic data by replacing the person’s name and surname with the respective universal identification code (IDUNI).

The physician reviewed the paper medical record, and entering data into the ARS application will take care to use the IDUNI code corresponding to the individual patient enrolled.

The anonymized databases were sent to ARS via a protected channel specifically built by the Information and Communications Technology Operational Unit of the ARS.

The pseudonymization procedure allowed ARS to carry out the record linkage operation between the databases, making it possible to build a clinical and symptomatic continuum between hospitalization and follow-up while respecting the patient’s privacy.

### 2.2. Statistic Analysis

A descriptive analysis of the outcomes (severity of pathologies and complications) and the main risk factors considered (age, gender, BMI, tobacco consumption, comorbidities) is carried out. For risk factors, the crude and adjusted relative risk (with a 95% confidence interval) of the individual outcomes for the effect of age, gender, and any other confounding risk factors was calculated. The estimate of the adjusted relative risks was calculated using regression models (logistic, linear, or Poisson, depending on the type of outcome considered).

## 3. Results

### 3.1. Primary Endpoint: Evaluate the Features of a COVID-19 Cohort at Hospital Admission in 2020 and 2021

#### Socio-Demographic and Clinical Description of the Cohort

The population enrolled in the index period (1 March 2020–1 March 2021) carried out at least 1 follow-up visit within 12 months after discharge was constituted of 2888 patients, of whom 58.5% are males. Overall, the average age is 66.2 years, with a slight difference between the two sexes, which identifies females as older (Table 2). The average age of the deceased was 82 years. Analyzing in-hospital mortality alone, it can be observed that this represented approximately 20% of the overall discharges but, among the elderly (class of age > 70 years), reached around 50%. The average age per hospitalization period is 65.3 years in the first phase and 66.5 years in the second one (Table 2).

Analyzing by month of hospitalization, the COVID-19 patients were enrolled mainly in March–April 2020 but above all in the second wave, which reached its peak in February 2021 (Table 3).

The laboratory parameters of the study population for a total of 1577 patients were analyzed, associated with to the Pisana and Senese University Hospitals, and Center and South East Local Hospitals. The inflammation indices, white blood cells and liver enzymes are well above the normal range. Upon hospital discharge, the tests show a significant difference compared with hospital admission, although many tests are still above the normal range (Supplementary Table S1).

Although most patients had undergone radiological examinations, few investigations were reported according to the classification by D’Andrea et al., 2020 [11]. Out of 86 subjects with clinical reports, 22 had results of previous pneumonia from SARS-CoV-2, one subject had an early form, 39 had a mild form, 7 had a severe form of the disease, and 17 reports were within limits. Since the sample size was so small, it was impossible to carry out supplemental analyses.

### 3.2. Secondary Endpoint: Identify the Factors Capable of Influencing COVID-19 Severity and Prognosis of Hospitalized Patients in Tuscany for SARS-CoV-2 Infection

Out of 2888 patients enrolled, 413 critical COVID-19 patients (14.3%) were admitted to an intensive care unit. No differences related to sex were observed (14.7% of males and 13.8% of females) while, as regards age, the selection gap due to the high in-hospital mortality of the elderly and the low number of places of intensive care, which characterized that pandemic period, can explain the greater use of high-intensity care departments in the younger age groups (Table 4). The use of NIV in critical COVID-19 patients showed a prevalence of 14.4%.

Considering the total number of patients for whom the field regarding tobacco use was filled in (n = 1536), the percentage of smokers represents 25% (Table 5).

As regards body weight, by applying the arithmetic calculation (weight/height^2^) to the values detected during hospital admission, the enrolled population was divided into underweight (BMI < 19 kg/height), normal weight (BMI 19–24 kg/height^2^), overweight (BMI 25–30 kg/height^2^), and obese (BMI > 30 kg/height^2^). As in the case of tobacco smoking, also in this case, not all the medical records of participating patients contained a specific field on body weight (67% of the total), but the data collected show a high prevalence of patients belonging to the overweight and obese people who represent approximately 65% of the total. In particular, in men, this value exceeds 68% even if, among women, the percentage of obese people is slightly higher (Table 6).

The analysis showed an NIV prevalence of 12.8% (CI 11–14.8) in the group of overweight and obese patients, while in the underweight and normal weight group, it was 10.2% (CI 8.2–12.6), highlighting a slight difference but not statistically significant (*p* = 0.083).

In the study population in 2020 vs. 2021, the main pharmacotherapy (Supplementary Table S2) used were systemic corticosteroids (19.3% vs. 51.9%), drugs for acidity-related disorders (27.3% vs. 42%), antibiotics (46.7% vs. 20%), and antithrombotics (27% vs. 19.1%). Pharmacotherapy (Supplementary Table S2) refers to the two periods (2020 and 2021) considered for hospitalization for COVID-19.

In a subgroup of patients (n = 489) associated with Pisa University Hospital, by dividing patients with and without alterations in AST (aspartate aminotransferase) and/or ALT (alanine aminotransferase) and/or gamma-GT (gamma-glutamyl transpeptidase) (at least higher than normal values), the association with worse outcomes (such as NIV, considered as the dependent variable) was studied (Table 7 and Table 8). The alteration of liver enzymes was associated with NIV (aOR = 2.28).

Our results confirm a greater risk of undergoing NIV or being admitted to intensive care in overweight (RR = 1.30, *p*-value = 0.022) and obese subjects (RR = 1.62, *p*-value < 0.001) (Supplementary Table S3). By adjusting the estimates for age, the strength of the association tends to decrease, also due to the decrease in statistical power. However, the adjustment for sex and age shows a persistence of significance for all obese subjects regardless of sex.

Regarding smoking, our study found a strong correlation between negative outcomes (NIV and/or intensive care) in smoking (RR = 4.91) or ex-smoking (RR = 3.48) patients hospitalized for COVID-19, and the strong association remains even when adjusted for age, gender and other factors.

In the entire patient cohort, 31.8% had at least one comorbidity (supplementary Table S4). Among the comorbidities, the most frequent was arterial hypertension (14.4%). Furthermore, subjects with comorbidities are also at greater risk of a more severe evolution towards critical COVID-19 (adjusted RR = 1.38). Among the pathologies most associated with negative outcomes, we found arterial hypertension (Supplementary Table S3). However, only a few patients (N = 16) had three comorbidities (Supplementary Table S5).

## 4. Discussion

The entire cohort of our study represented the COVID-19 *surviving people*. The average age of this cohort is lower than that observed in the population infected in the same period due to the high mortality recorded among the so-called elderly population. During the two pandemic waves covered by our study, the average age of the deceased was 82 years. The study population showed a higher prevalence of women than men, as our cohort included COVID-19 survivors. Several studies [13,14] have demonstrated that COVID-19 is more lethal among males than females; therefore, we hypothesized that women were prevalent in our study, as many more men died during hospitalization. Contrary to other studies [15,16], the prevalence of smokers among those hospitalized for COVID-19 appears lower than expected. Recent systematic reviews carried out on the same topic indicate a greater risk of mortality among smokers and former smokers hospitalized for COVID-19 compared to never-smokers [17]. An Italian multi-center longitudinal study by Gallus et al. [18] involving 24 Italian hospitals for a total of 1820 laboratory-confirmed COVID-19 patients demonstrated a dismal COVID-19 progression in current smokers and second-hand smoke-exposed non-smokers. The discrepancy in our results is probably due to the low number of subjects considered in the analysis due to missing values for the specific field.

Our cohort showed a high prevalence of patients belonging to overweight and obese categories (65%), and our results confirmed a greater risk for overweight or obese subjects of NIV or admission to intensive care. The importance of body weight in evaluating the course and outcomes of SARS-CoV-2 infection has been strongly demonstrated in several studies [19,20]. According to our findings, a cohort study conducted in 2020 in Seattle showed that COVID-19 patients requiring mechanical ventilation had an average BMI of 33 kg/m^2^ [21]. Chetboun et al. [22] conducted an international multicenter retrospective cohort study, confirming an association between BMI and invasive mechanical ventilation in patients with COVID-19, also observing higher values in younger women independent of the presence of other metabolic risk factors [22]. The relevance of body weight is due to the mechanism of penetration of the SARS-CoV-2 virus in human cells, i.e., the binding to the ACE2. The involvement of the ACE2 receptor in the viral replication system also raises concerns about the use of ACE inhibitor drugs in the treatment of hypertension. Among the main theories of the increased mortality observed among patients with COVID-19 treated with ACE inhibitors and angiotensin receptor beta blockers, the intake of antihypertensive drugs was hypothesized to increase the expression of ACE2, negatively affecting the severity and evolution of the disease. In hypertensive patients with COVID-19 taking ACEIs or angiotensin receptor blockers, Kerneis et al. [23] showed that the mortality rate was significantly low, suggesting that these drugs should not be discontinued. In obese subjects, the condition of insulin resistance is associated with altered functioning of the renin-angiotensin-aldosterone system (adipose tissue favors the production of factors capable of increasing the production of angiotensinogen and aldosterone) [24], which causes a high expression of ACE2 in adipose tissue cells constituting privileged access to the SARS-CoV-2 virus. In addition, in obese patients, the difficulties of ventilatory mechanics were due to the decreased respiratory volume and the respiratory compromise in the supine position due to the reduced diaphragmatic excursion [25].

Worse outcomes of COVID-19 have been found in the elderly population and subjects suffering from other comorbidities, consisting of a greater risk of hospitalization, access to intensive care units, and mortality. In the entire patient cohort, 31.8% had at least one comorbidity, and among the comorbidities, the most frequent was arterial hypertension, which in turn was associated with negative outcomes.

Among the possible interpretations, we must highlight that uncontrolled higher systolic blood pressure may contribute to a more severe course of the disease due to its association with greater vascular stiffness, including vascular remodeling, which may worsen endothelial dysfunction and damage, induced by SARS-CoV-2 infection [26].

Several clinical trials and retrospective studies were carried out on cohorts of patients hospitalized for COVID-19 [27]. Recently, a meta-analysis conducted on sixteen randomized controlled clinical trials suggests the maintenance of therapy with renin-angiotensin-aldosterone system (RAAS) inhibitors in patients with non-severe COVID-19, where indicated, on the contrary, the initiation of RAAS treatment could be harmful in critically ill patients [28]. In our cohort, the use of RAAS was 10% in the first period and 5.9% in the second. In the entire study period (2020 and 2021), the most used drugs in our cohort were corticosteroids, antacids, antibiotics, and antithrombotics. Regarding the analysis of the therapeutic regimen, two different periods were compared. The first 10 months (March–December 2020) were compared to 3 months (January–March 2021). The difference between these two periods was due not only to the length of the period but also to the different knowledge of the pathogenesis of COVID-19. The first period differed from the second one for the lesser knowledge of the characteristics of the COVID pathology, the lack of availability of the anti-SARS-CoV-2 vaccine, and the circulation of different viral variants, reflected in the use of therapeutic regimes.

Thrombotic risk caused by COVID-19, currently demonstrated by several studies, only five months after the start of the COVID-19 pandemic (May 2020), was not sufficiently documented, representing a clinical suspicion awaiting confirmation [29]. The same difficulty had been encountered in the association between COVID-19 severity and the presence of clinical, laboratory, and radiological features, which, in turn, were subject to continuous updates necessary to allow evidence-based clinical practice [30]. Heparin, currently included in the COVID-19 treatment protocols, was intended for use following an accurate assessment of the thrombotic risk [31]. The use of antithrombotics in our cohort maintained widespread usage in both 2020 and 2021 (27% vs. 19%) and indirectly suggests risk for thrombotic complications.

Recent studies confirm the maintenance of high values of the D-dimer level up to 6 months after discharge. Persistence of elevated D-dimer levels is associated with short- and long-term sequelae [32]. In fact, in SARS-CoV-2 infection, the coagulation pathway is activated by the immune response and cytokine storm, which leads to a hypercoagulable and hyperinflammatory state [33]. Red blood cells could also play a role in the cytokine storm, as they store and release several cytokines, including TNF-α and IL-1β, which are pro-inflammatory [34].

Contrary to other studies [35], our data did not highlight a significant association between the presence of diabetes and a greater risk for negative outcomes. This finding could be due because this is a cohort of subjects who survived the acute form of COVID-19, whereas diabetes was associated with a greater risk of COVID-19 mortality. The presence of a few comorbidities in our cohort could be explained in the same way. Apart from a greater number of comorbidities that suggest a possible major frailty, we do not have other clinical data that could allow us to calculate the frailty index in these patients. In a clinical study [36] of 729 patients, frailty was assessed according to the Clinical Frailty Scale. This study demonstrated that severe disease, the presence of ≥3 comorbidities, male sex, and frailty were independent risk factors for in-hospital death. Despite the limitations of our study, which did not evaluate frailty, as the clinical data necessary for calculating the score were not present, it conversely showed that in the cohort of survivors, comorbidities are lower, as is the prevalence of male sex and old age.

The treatment of patients with COVID-19 also ranged from symptomatic treatment to ventilatory support based on the severity of lung involvement. The first therapeutic approach also saw the use of Hydroxychloroquine sulfate, on which the Food and Drug Administration expressed its opinion in June 2020, revoking its emergency use due to the serious cardiac consequences induced by the treatment. The corticosteroid use was subsequently approved by the World Health Organization (WHO) only following the publication of results from multicenter studies [37]. In our cohort, the use of corticosteroids reached 51.9% in 2021 compared to 19.1% in 2020, probably for a more accredited effectiveness demonstrated by clinical studies and for the approval of the WHO in this context.

The limits of our study were represented by the difficulties encountered in the implementation of a regional database acting to follow the entire evolution of the clinical picture (e.g., evolution/regression of pulmonary, cardiovascular, and renal involvement linked to the initial COVID-19 severity) on a representative number of patients. Furthermore, many limitations of the analyses carried out are linked to the missing values of some variables, such as those relating to transaminase values.

This study in a large cohort of patients demonstrated the risk for non-invasive ventilation in patients with AST and/or ALT and/or gamma-GT higher than normal values.

The limitation of this finding is that the role of a pre-existing chronic liver disease linked to hepatitis viruses or other causes, such as alcohol-induced fatty liver disease, obesity, diabetes, or a combination of clinical conditions (metabolic syndrome), as a predisposing factor of this alteration, cannot be excluded. It should be noted that these patients underwent potentially hepatotoxic pharmacological therapies during hospitalization. A systematic review [38] for a total of 57 studies, of which 33 (57.9%) were from China, forty-two of the 57 studies reported abnormalities in liver enzymes, and six studies indicated reactivation of HBV. This finding in the Chinese population could be due to the very high endemicity of HBV infection in that population compared to other countries, including Italy [38].

Righi et al. [39] describe the histological characteristics of the liver in a large cohort of deceased patients with COVID-19 compared to a control group of deceased patients without COVID-19 who underwent autopsy. The livers of COVID-19-positive patients, excluding those with alcoholic liver disease, had mild lobular inflammation and steatosis, suggesting that steroid use, at least in part, may be responsible for the increased steatosis in the COVID-19-positive group, which can also worsen the underlying non-alcoholic steatohepatitis. Romano et al. [40], in an Italian retrospective study of 123 patients admitted to COVID-19 centers between the end of 2020 and the spring of 2021, dividing patients into two groups (with normal liver biochemistries vs. altered liver function tests), demonstrated that a higher prevalence of patients with liver enzyme alterations (74% vs. 65%) develop COVID-19 pneumonia. These kinds of patients needed more days of respiratory support and intensive administration of supplemental oxygen, suggesting that liver abnormalities at admission could be predictive of more severe interstitial pneumonia due to COVID-19. Poudel et al. [41] showed in a cohort of English patients that the presence of liver injury in patients with COVID-19 infection at the time of hospital admission was an independent predictor of poor outcomes and disease severity.

## 5. Conclusions

Our cohort is comprised of overweight and obese 65% of patients, with a mean age of 66.2 years. Smokers/ex-smokers, overweight/obese subjects, and comorbidities were at higher risk of adverse outcomes. Moreover, a significant association was found between the alterations of liver enzymes and worse outcomes. The results provide valuable insights into the clinical and epidemiological features of COVID-19 patients.

We characterized this patient population because, in our future studies, we are evaluating the short- and long-term regression/evolution of the specific clinical manifestations of SARS-CoV-2 infection in patients hospitalized in Tuscany and the persistence of symptoms compatible with COVID-19 and the psycho-emotional impact in a subgroup of patients discharged from hospital.

## Figures and Tables

**Table 1:** Main information collected during the study period.

Variable	
**Socio-demographic**	First name; Surname; Date of birth; Educational qualification (if available); Smoking (if available); Weight (for BMI calculation); Height
**Clinical**	
Pneumological	Noninvasive mechanical ventilation
**Comorbidities**	**ICDIX-Cm codes**
Blood samples	Hematocrit; Hemoglobin; White blood cells; Lymphocytes; Lymphocytopenia; Platelets; Transaminases; gamma glutamyl transpeptidase; Creatinine; C-reactive protein; Albumin; Azotemia; Ferritin; D Dimer.

**Table 2:** Subjects (n and %) hospitalized for COVID-19 in the period 1 March 2020–1 March 2021.

Age Class at Admission	Females		Males		Total
	n	%	n	%	n
18–19 years	2	0.2	1	0.1	3
20–29 years	18	1.5	10	0.6	28
30–39 years	30	2.5	45	2.7	75
40–49 years	99	8.3	142	8.4	241
50–59 years	190	15.8	373	22.1	563
60–69 years	261	21.8	460	27.2	721
70–79 years	307	25.6	422	25.0	729
80–89 years	226	18.8	217	12.8	443
90+ years	66	5.5	19	1.1	85
Total	1.199	100.0	1.689	100.0	2.888
Mean age	Mean	CI95%	Mean	IC95%	Mean
	67.9	(64.4–65.6)	65.0	(67.0–68.7)	66.2

Patients carried out at least one follow-up visit in the 12 months following discharge—Analysis by sex and age class—Source: ARS on Sprint data.

**Table 3:** Subjects (n and %) hospitalized for COVID-19 in the period 1 March 2020–1 March 2021.

Month	2020		2021		Total
	n	%	n	%	n
January	-	-	259	9.0	259
February	2	0.1	227	7.9	229
March	512	17.7	60	2.1	572
April	146	5.1	2	0.1	148
May	24	0.8	-	-	24
June	7	0.2	-	-	7
July	7	0.2	-	-	7
August	15	0.5	-	-	15
September	59	2.0	-	-	59
Ottobre	506	17.5	-	-	506
November	770	26.7	-	-	770
December	292	10.1	-	-	292
Total	2340	81.0	548	19.0	2888

Patients carried out at least one follow-up visit in the 12 months after discharge—Analysis of the month of hospitalization—Source: ARS on Sprint data.

**Table 4:** Subjects (n and %) hospitalized in intensive care for COVID-19 in the period 1 March 2020–1 March 2021.

Age Class at Admission	n	%
18–19 years	-	-
20–29 years	6	21.4
30–39 years	9	12.0
40–49 years	37	15.4
50–59 years	94	16.7
60–69 years	103	14.3
70–79 years	105	14.4
80–89 years	50	11.3
90+ years	9	10.6
Total	413	14.3

Patients carried out at least one follow-up visit in the 12 months following discharge—Analysis by age class—Source: ARS on Sprint data.

**Table 5:** Tobacco use for subjects (n and %) hospitalized for COVID-19 infection in the period 1 March 2020–1 March 2021.

	Ex Smoker		Smoker		Never Smoker		Total
	n	%	n	%	n	%	n
Gender							
Males	308	33.8	210	23.0	394	43.2	912
Females	121	19.4	173	27.8	330	52.8	624
Age							
18–19 years	1	100.0	-	-	-	-	1
20–29 years	1	6.7	9	60.0	5	33.3	15
30–39 years	4	13.8	10	34.5	15	51.7	29
40–49 years	27	23.1	39	33.3	51	43.6	117
50–59 years	71	25.4	85	30.4	124	44.3	280
60–69 years	125	30.0	105	25.2	187	44.8	417
70–79 years	126	32.6	85	22.0	176	45.5	387
80–89 years	69	28.2	41	16.7	135	55.1	245
90+ years	5	11.1	9	20.0	31	68.9	45
Total	429	27.9	383	25.0	724	47.1	1.536

Patients carried out at least one check-up visit in the 12 months following discharge—Analysis by gender and age groups—Source: ARS on Sprint data.

**Table 6:** Weight categories (based on BMI calculation) in subjects (n and %) hospitalized for COVID-19 in the period 1 March 2020–1 March 2021.

BMI	Males		Females		Total
	n	%	n	%	n
Underweight	11	1.0	19	2.3	30
Normal weight	335	30.3	309	37.2	644
Overweight	514	46.6	307	37.0	821
Obese	244	22.1	195	23.5	439
Total	1104	100.0	830	100.0	1934

Patients carried out at least one follow up visit in the 12 months following discharge—Gender analysis—Source: ARS on data Sprint.

**Table 7:** Liver enzymes in the study population and association with non-invasive mechanical ventilation.

Alteration of Liver Enzymes *	NIV				Total	
	No		Yes			
	No	%	n	%	n	%
no	321	81.1 (76.9–84.6)	75	18.9 (15.4–23.1)	396	100.0
yes	61	65.6 (55.4–74.6)	32	34.4 (25.5–44.6)	93	100.0
Total	382	78.1 (74.2–81.6)	107	21.9 (18.4–25.8)	489	100.0

* at least higher than normal values. Normal range: AST ≤ 45 U/L; ALT ≤ 45 U/L; gammaGT ≤ 60 U/L; NIV, Noninvasive mechanical ventilation.

**Table 8:** Crude and sex and age adjusted logistic regression.

	OR	INF	UPPER	*p*-Value
OR crude	2.25	1.37	3.69	0.001
OR adj sex and age	2.28	1.36	3.83	0.002

## Data Availability

The clinical and laboratory data are presented in this study.

## Supplementary Materials

The following supporting information can be downloaded at: https://www.mdpi.com/article/10.3390/jcm13164626/s1, Table S1: Laboratory parameters in the study population. Table S2: Pharmacotherapy in the study population. Table S3. Relative risks (RR) for patients admitted to intensive care/NIV versus those not admitted to intensive care/NIV—analysis of the main risk factors - Source: ARS on Sprint data. Table S4. Comorbidities in patients admitted to intensive care/NIV versus those not admitted to intensive care/NIV—Source: ARS on Sprint data. Table S5. Comorbidities in patients admitted to intensive care/NIV versus those not admitted to intensive care/NIV—Source: ARS on Sprint data.
