# Supplementary material for: Retrospective Study on the Features and Outcomes of a Tuscany COVID-19 Hospitalized Patients Cohort: Preliminary Results
Source: J Clin Med. 2024 Aug 7;13(16):4626. doi: 10.3390/jcm13164626 (PMC11354555; doi:10.3390/jcm13164626)
Supplement: Supplementary file 1 [file jcm-13-04626-s001.zip › jcm-3103046-supplementary.pdf]

Supplementary Table S1. Laboratory parameters in the study population

Blood tests	N	Mean ±SD at admission	% above threshold	Mean ±DS at discharge	% above threshold	difference	p-value and paired t-test
**WHITE BLOOD CELLS (10^9/L)**	1.011	7.8 ± 4.0	21	8.0±3.6	19.5	-0.2	0.109
**RED BLOOD CELLS (10^12/L)**	1.554	4.5 ± 0.7	3.7	4.2±0.6	1.8	0.2	0.000
**HEMOGLOBIN (G/DL)**	1.557	13.0 ± 1.8	1.8	12.3±1.7	0.6	0.7	0.000
**HEMATOCRIT (%)**	1.557	38.9 ± 5.0	4.2	37.2±4.9	1.9	1.7	0.000
**PLATELETS (10^9/L)**	1.450	229.4 ± 90.4	2.6	279.2±116.9	8.0	-49.8	0.000
**ALBUMIN (G/DL)**	411	46.4± 355.6	1.9	55.6±401.1	1.7	-9.2	0.269
**ALT (U/L)**	993	43.4± 62.6	30	56.2±52.1	48.6	-12.8	0.000
**AST (U/L)**	934	38.6 ±56.6	26.2	28.3±19.4	15.6	10.3	0.000
**AZOTEMIA (MG/DL)**	867	47.8 ±29.8	31	45.7±27.9	26.2	2.1	0.007
**D DIMER (MG/L)**	1.061	1.4 ± 5.6	56	1.1±4.7	56	0.3	0.127
**FERRITIN (NG/ML)**	584	838.0 ±790.7	74.8	689.0±578.0	73.8	149.0	0.000
**GAMMAGT (U/L)**	627	84.4 ± 156.8	39.7	79.9±96.7	41.6	4.5	0.363
**GFR (ML/MIN/1.TMQ)**	1.511	79.5 ±24.1	0	84.4±22.3	0	-5.0	0.000
**PROTEIN C (MG/DL)**	1.430	7.5 ± 6.9	93.2	1.7±2.9	54.3	5.8	0.000

Supplementary Table S2. Pharmacotherapy in the study population

	therapy in 2020	Total hospitalizations in 2020	therapy (%) in 2020	therapy in 2021	Total hospitalizations in 2021	therapy (%) in 2021
SYSTEMIC CORTICOSTEROIDS	94	488	19.3	691	1.332	51.9
ANTIACIDS	133	488	27.3	559	1.332	42.0
ANTIBACTERIALS FOR SYSTEMIC USE	228	488	46.7	266	1.332	20.0
ANTITHROMBOTICS	132	488	27.0	255	1.332	19.1
ANALGESICSI	94	488	19.3	196	1.332	14.7
BLOOD SUBSTITUTES AND PERFUSION SOLUTIONS	97	488	19.9	185	1.332	13.9
PREPARATIONS FOR COUGH AND COLD ILLNESSES	37	488	7.6	132	1.332	9.9
DRUGS USED IN DIABETES	62	488	12.7	112	1.332	8.4
ALL OTHER THERAPEUTIC PRODUCTS	24	488	4.9	89	1.332	6.7
PSYCHOLEPTICS	59	488	12.1	87	1.332	6.5
DRUGS FOR CONSTIPATION	40	488	8.2	85	1.332	6.4
SUBSTANCES ACTING ON THE RENIN-ANGIOTENSIN SYSTEM	49	488	10.0	78	1.332	5.9
DIURETICS	35	488	7.2	71	1.332	5.3
DRUGS FOR OBSTRUCTIVE AIRWAY DISORDERS	29	488	5.9	61	1.332	4.6
CALCIUM ANTAGONISTS	74	488	15.2	57	1.332	4.3
ANTIVIRALS FOR SYSTEMIC USE	98	488	20.1	53	1.332	4.0
LIPIDS MODIFYING SUBSTANCES	20	488	4.1	51	1.332	3.8
CARDIAC THERAPY	10	488	2.0	50	1.332	3.8
BETA BLOCKERS	64	488	13.1	49	1.332	3.7
PSYCHOANALEPTICS	31	488	6.4	46	1.332	3.5
VITAMINS	27	488	5.5	41	1.332	3.1
MINERAL SUPPLEMENTS	69	488	14.1	35	1.332	2.6
ANTIDIARRHOIC, ANTI-	21	488	4.3	27	1.332	2.0

INFLAMMATORY AND INTESTINAL ANTIMICROBIAL						
THYROID THERAPY	27	488	5.5	24	1.332	1.8
UROLOGICALS	21	488	4.3	20	1.332	1.5
DRUGS FOR GASTROINTESTINAL FUNCTION DISORDERS	29	488	5.9	20	1.332	1.5
ANTIANEMIC DRUGS	17	488	3.5	14	1.332	1.1
ANESTHETICS	11	488	2.3	12	1.332	0.9
ANTIGUTTOSIS	14	488	2.9	12	1.332	0.9
ANTIHYPERTENSIVES	13	488	2.7	11	1.332	0.8
ANTI-INFLAMMATORY AND ANTIRHEUMATIC DRUGS	12	488	2.5	9	1.332	0.7
OPHTHALMOLOGISTS	12	488	2.5	9	1.332	0.7
IMMUNOSUPPRESSORS	17	488	3.5	9	1.332	0.7
MIORELAXANTS	8	488	1.6	9	1.332	0.7
ANTIEPILEPTICS	8	488	1.6	8	1.332	0.6
CYTOSTATIC	1	488	0.2	7	1.332	0.5
OTHER PREPARATIONS FOR THE RESPIRATORY SYSTEM	1	488	0.2	6	1.332	0.5
ANTIHEMORRHAGIC	1	488	0.2	6	1.332	0.5
ANTIHISTAMINES FOR SYSTEMIC USE	4	488	0.8	6	1.332	0.5
ANTIFUNGALS FOR SYSTEMIC USE	7	488	1.4	5	1.332	0.4
ALTERNATIVE DESCRIPTION	0	488	0.0	4	1.332	0.3
OTHER NERVOUS SYSTEM DRUGS	1	488	0.2	4	1.332	0.3
ANTIPARKINSONIANS	2	488	0.4	3	1.332	0.2
ANTIEMETICS AND ANTINAUSEA	2	488	0.4	2	1.332	0.2
ENDOCRINE THERAPY	2	488	0.4	2	1.332	0.2
ANTIBIOTICS AND CHEMOTHERAPIES FOR DERMATOLOGICAL USE	0	488	0.0	2	1.332	0.2
VASOPROTECTORS	0	488	0.0	1	1.332	0.1
ANTIPROTOZOARIANS	80	488	16.4	1	1.332	0.1
HOMEOPATHIC UNITARIES	0	488	0.0	1	1.332	0.1
BILIARY AND LIVER THERAPY	1	488	0.2	1	1.332	0.1
PITUITARY, HYPOTHALAMIC AND ANALOGUE HORMONES	0	488	0.0	1	1.332	0.1
SEX HORMONES AND MODULATORS OF THE GENITAL SYSTEM	1	488	0.2	1	1.332	0.1
DENTISTRY	2	488	0.4	0	1.332	0.0

ALL OTHER NON-THERAPEUTIC PRODUCTS	1	488	0.2	0	1.332	0.0
DIGESTIVES, INCLUDING ENZYMES	1	488	0.2	0	1.332	0.0

Supplementary Table S3. Relative risks (RR) for patients admitted to intensive care/NIV versus those not admitted to intensive care/NIV - analysis of the main risk factors - Source: ARS on Sprint data

Variable	Level	Raw RR				RRs adjusted for age and gender				RRs adjusted for age, gender, BMI, smoking and chronicity			
		RR	Min	Max	p-value	RR	Min	Max	p-value	RR	Min	Max	p-value
If yes	Male	1.00				1.00				1.00			
	Female	0.70	0.61	0.82	0.000	0.76	0.66	0.88	0.000	0.84	0.70	1.02	0.082
CI age axis	18-49	1.00				1.00				1,00			
	50-59	1,11	0,88	1,40	0,386	1,09	0,86	1,37	0,484	1,17	0,85	1,62	0,334
	60-69	1,19	0,96	1,49	0,118	1,18	0,95	1,48	0,133	1,13	0,83	1,55	0,441
	70-79	0.94	0.75	1.19	0.624	0.94	0.75	1.19	0.624	0.90	0.64	1.26	0.537
	80+	0.41	0.30	0.56	0.000	0.42	0.31	0.58	0.000	0.57	0.37	0.89	0.012
At least 1 chronic disease	No	1.00				1.00				1.00			
	Yes	1.13	0.99	1.31	0.080	1.20	1.04	1.38	0.014	1.38	1.15	1.65	0.000
Cardiac diseases	No	1.00				1.00							
	Yes	0.93	0.70	1.22	0.578	1.02	0.78	1.34	0.861				
Diabetes	No	1.00				1.00							
	Yes	1.08	0.87	1.35	0.489	1.06	0.85	1.32	0.591				
BMI	Normal weight/underweight	1.00				1.00				1.00			
	Overweight	1.30	1.04	1.63	0.022	1.20	0.96	1.49	0.116	1.11	0.89	1.38	0.350
	Obese	1.62	1.28	2.07	0.000	1.39	1.09	1.77	0.007	1.14	0.90	1.45	0.286
Smoke	No	1.00				1.00				1.00			
	Ex smoker	3.48	2.67	4.53	0.000	3.33	2.56	4.33	0.000	3.43	2.59	4.55	0.000
	Smoker	4.91	3.82	6.31	0.000	4.61	3.59	5.93	0.000	4.54	3.47	5.96	0.000
Hypertension	No	1.00				1.00							
	Yes	1.28	1.08	1.52	0.00	1.29	1.09	1.53	0.00				

					5				4				
Neurological diseases	No	1.00				1.00							
	Yes	0.77	0.41	1.45	0.423	1.23	0.65	2.34	0.529				
BPCO	No	1.00				1.00							
	Yes	0.81	0.54	1.22	0.312	0.86	0.57	1.30	0.486				
Oncological diseases	No	1.00				1.00							
	Yes	1.12	0.74	1.68	0.593	1.14	0.77	1.70	0.520				

Supplementary Table S4.Comorbidities in **patients admitted to intensive care/NIV versus those not admitted to intensive care/NIV - Source: ARS on Sprint data**

	no NIV/TI			in NIV/TI			**Total**		
	%	min	max	%	min	max	%	**min**	**max**
At least 1 chronic disease	31.0	29.0	32.9	34.6	30.9	38.2	**31.8**	**30.1**	**33.5**
BPCO	3.8	3.0	4.6	2.9	1.6	4.2	**3.6**	**2.9**	**4.3**
Cardiac diseases	7.3	6.2	8.4	6.6	4.7	8.6	**7.1**	**6.2**	**8.1**
Diabetes	9.3	8.1	10.5	10.2	7.9	12.5	**9.5**	**8.4**	**10.6**
Hypertension	13.5	12.1	14.9	17.7	14.8	20.7	**14.4**	**13.2**	**15.7**
Neurological diseases	1.7	1.2	2.2	1.2	0.4	2.1	**1.6**	**1.1**	**2.1**
Oncological diseases	2.4	1.8	3.0	2.8	1.5	4.0	**2.5**	**1.9**	**3.1**

Supplementary Table S5. Comorbidities in patients admitted to intensive care/NIV versus those not admitted to intensive care/NIV - Source: ARS on Sprint data

**Number of chronic diseases**	**no NIV/TI (n: 2.239)**				**in NIV/TI (n: 648)**				**Total (n: 2.887)**			
	**n**	**%**	**min**	**max**	**n**	**%**	**min**	**max**	**n**	**%**	**min**	**max**
0	1.546	69.1	67.1	70.9	424	65.4	61.7	69.0	1.970	68.2	66.5	69,9
1	552	24.7	22.9	26.5	181	27.9	24.6	31.5	733	25.4	23.8	27,0
2	125	5.6	4.7	6.6	41	6.3	4.7	8.5	166	5.8	5.0	6,7
3	16	0.7	0.4	1.2	2	0.3	0.1	1.2	18	0.6	0.4	1,0
Total	2.239	100			648	100			2.887	100		
